# Role of necroptosis and immune infiltration in preeclampsia: novel insights from bioinformatics analyses

**DOI:** 10.1186/s12884-023-05821-0

**Published:** 2023-07-04

**Authors:** Lidan He, Feng Zhan, Lin Lu, Xia Zhang, Jianbo Wu

**Affiliations:** 1 Department of Obstetrics and Gynecology, The First Affiliated Hospital of Fujian Medical University, Fujian, 350004 China; 2 College of Engineering, Fujian Jiangxia University, Fuzhou, 350108 China

**Keywords:** Preeclampsia, Necroptosis, Immune Infiltration, Bioinformatics

## Abstract

**Background:**

Preeclampsia (PE) is a serious pregnancy complication that can adversely affect the mother and fetus. Necroptosis is a recently discovered new form of programmed cell death involved in the pathological process of various pregnancy complications. Our study aimed to identify the necroptosis-related differentially expressed genes (NRDEGs), create a diagnosis model and related disease subtypes model based on these genes, and further investigate their relationship with immune infiltration.

**Methods:**

In this study, we identified NRDEGs by analyzing data from various databases, including Molecular Signatures, GeneCards, and Gene Expression Omnibus (GEO). Using minor absolute shrinkage and selection operator (LASSO) and logistic Cox regression analysis, we developed a novel PE diagnosis model based on NRDEGs. Furthermore, we developed PE subtype models using consensus clustering analysis based on key gene modules screened out by weighted correlation network analysis (WGCNA). Finally, we identified the difference in immune infiltration between the PE and control groups as well as between both PE subtypes by analyzing the immune cell infiltration across combined datasets and PE datasets.

**Results:**

Our study discovered that the necroptosis pathway was significantly enriched and active in PE samples. We identified nine NRDEGs that involved in this pathway, including BRAF, PAWR, USP22, SYNCRIP, KRT86, MERTK, BAP1, CXCL5, and STK38. Additionally, we developed a diagnostic model based on a regression model including six NRDEGs and identified two PE subtypes: Cluster1 and Cluster2, based on key module genes. Furthermore, correlation analysis showed that the abundance of immune cell infiltration was related to necroptosis genes and PE disease subtypes.

**Conclusion:**

According to the present study, necroptosis is a phenomenon that occurs in PE and is connected to immune cell infiltration. This result suggests that necroptosis and immune-related factors may be the underlying mechanisms of PE pathophysiology. This study opens new avenues for future research into PE's pathogenesis and treatment options.

**Supplementary Information:**

The online version contains supplementary material available at 10.1186/s12884-023-05821-0.

## Background

Preeclampsia (PE), a pregnancy-specific cardiovascular problem that affects 2 to 8% of women and is a substantial cause of maternal and fetal mortality, is defined by newly developed hypertension after 20 weeks of gestation, along with proteinurias or multiple organ injuries, such as renal insufficiency, liver dysfunction, cerebral or visual disturbances, and edema [1]. Although, during the past few decades, considerable research has been done on the causes of PE. However, its etiology and pathology are still poorly understood, which remains a severe challenge in obstetrics [2]. Hypertension control and maternal–fetal monitoring have limited therapeutic effects; the only effective treatment for PE is still timely pregnancy termination [3]. Early identification, precise diagnosis, and effective therapy of PE are crucial to lowering the risk of poor maternal–fetal outcomes. Therefore, finding new possible biomarkers to screen for, diagnose, and track PE is essential.

The pathogenesis of PE is complex and involves various mechanisms, such as immunological dysregulation, damage to the vascular endothelial cell, and hyperinflammatory response [4], etc. The predominant view is that aberrant trophoblast cell invasion might result in inadequate remodeling of the maternal spiral arteries, which would cause uteroplacental high resistance circulation, and placental ischemia and hypoxia [5]. In recent years, numerous types of research have shown that necroptosis plays a vital role in trophoblast injury and placental physiology [6, 7] and that necroptosis is distinct from pyroptosis and apoptosis [8].

Necroptosis is a recently discovered type of programmed cell death with hallmark necrosis characteristics [9]. It is a modulated necrotic cell death mediated by RIP1 and RIP3 kinases with both passive and active pro-inflammatory functions. Necroptosis combines several features of apoptosis and necrosis, such as membrane integrity loss, organelle swelling, cell lysis, intracellular component leaking, and death receptor ligand induction [10]. Numerous research has recently indicated that necroptosis is involved in the pathogenesis of cardiovascular illnesses [11, 12], tumors [13], inflammatory lesions [14], neurodegenerative diseases [15], etc. Recent research has demonstrated that necroptosis is crucial to the incidence and progression of PE [16]. Therefore, the identification of necroptosis-related biomarkers is essential for the early diagnosis of PE and helpful for the early intervention of patients with PE.

In the past ten years, immune infiltration has attracted much attention to cancer. Increasing research has indicated that immune inflammation is involved in the pathophysiology of PE [17, 18]. Numerous bioinformatic analyses also suggest the difference in immune infiltration between PE and conventional control [19]. However, little is known about the potential mechanisms of immune infiltration in PE. Given the significance of immune cell infiltration in the pathogenesis of PE, immune cell infiltration analysis is helpful in screening the key genes, which could be crucial in identifying molecular markers of PE subtypes and further precision treatment.

In this work, using the gene expression omnibus (GEO) database, we used comprehensive bioinformatics analysis to explore the key genes and potential functional mechanisms of necroptosis in PE. Additionally, to comprehend the potential molecular immunity process during PE development, we overviewed the immune infiltration landscape and probed carefully into the connection between necroptosis and infiltrating immune cells.

## Materials and methods

### Data collection and processing

The PE-related GSE60438 dataset [20] was downloaded from the GEO database (https://www.ncbi.nlm.nih.gov/geo/) through the R package “GEOquery” [21]. Dataset samples were from decidua basalis of homo-sapiens, and the microarray platforms were GPL10558 and GPL6884, respectively. The GPL10558 contained 35 PE and 42 control samples, and the GPL6884 included 25 PE and 23 control samples. All PE and control samples used in the study are displayed in Table 1.

**Table 1:** GEO microarray chip information

	GSE60438	
Platform	GPL10558	GPL6884
Species	Homo sapiens	Homo sapiens
Tissue	Decidua basalis	Decidua basalis
Samples in PE Group	35	25
Samples in Control Group	42	23
Reference	PMID: 26010865	PMID: 26010865

*PE* Pre-eclampsia, *GEO* Gene Expression Omnibus

Necroptosis-related genes (NRGs) were collected from the GeneCards database [22] (https://www.genecards.org/) and Molecular Signatures Database (MSigDB) [23] (https://www.gsea- msigdb.org/gsea/msigdb/index.jsp). The GeneCards database provides comprehensive information on human genes. A total of 623 NRGs were obtained after using "Necroptosis" as the search keyword and keeping only "Protein Coding" in the GeneCards database. Similarly, eight NRGs were obtained by searching the MSigDB database with "Necroptosis" as the keyword necroptotic signaling pathway gene set contained. After combined deduplication, 623 NRGs were obtained, and the details are shown in Table S1.

The combined datasets were obtained by debatch-processing dataset GSE60438 by the R package “sva” [24], which contains 60 PE samples and 65 control samples. Finally, the combined GEO datasets were processed by the R package “limma” [25] to remove batch effects, standardize, annotate probes and normalize.

### Identification of the key necroptosis-related DEGs

We picked out differentially expressed genes (DEGs) using the R package “limma” (adjust *p*-value < 0.05 and | logFC (Fold Change) |> 0). Among them, adjust *p*-value < 0.05 and logFC > 0 were up-regulated DEGs, adjust *p*-value < 0.05 and logFC < 0 were down-regulated DEGs. The R packages “heatmap” and “ggplot2” were used to draw heat and volcano maps.

### Functional and pathway enrichment analysis

Gene Ontology (GO) [26] is a common method for large-scale functional enrichment studies, including cell composition (CC), biological process (BP) and molecular function (MF). Kyoto Encyclopedia of Genes and Genomes (KEGG) [27] is a widely used database containing information about genomes, biological processes, illnesses, and medications. GO and KEGG analyses of the NRDEGs were performed by the R package “ClusterProfiler” [28] and visualized by the R package “ggplot2”.

### Gene set enrichment analysis and gene set variation analysis

Gene Set Enrichment Analysis (GSEA) [29] is a calculational technique that assesses whether a specified set of genes displays statistically significant concordant differences between both biological states. GSEA was carried out in this study using the R package "ClusterProfiler." Each analysis procedure was performed 1000 times. The following parameters were utilized in the GSEA: seed 2020, 1000 computations, 10 minimum and 500 maximum number of genes in each gene set, and adjust *p*-value correction method of Benjamini-Hochberg (BH). The MSigDB database's "c2.cp.all.v2022.1.hs.symbols.gmt [all canonical pathways](3050)" gene set was utilized by GSEA for enrichment analysis. Statistically significant enrichment was defined as a function or pathway term with a false discovery rate (FDR) < 0.25 and adjust *p*-value < 0.05.

Gene Set Variation Analysis (GSVA) [30] is a non-parametric unsupervised analysis method that evaluates the gene set enrichment results of the microarray nuclear transcriptome by transforming the expression matrix of genes between different samples into the expression matrix of gene sets between samples to evaluate whether different pathways are enriched between different samples. Genes in combined datasets and genes in the "c2.all.v7.5.1.symbols.gmt" gene set were accessed via the MSigDB database. We estimated the difference in its functional enrichment between PE and control groups. |logFC|> 0.25 and an adjusted *p*-value of less than 0.05 were significantly enriched.

GSEA and GSVA analyses were used to identify rich biological pathways and gene sets in DEGs and key module genes, respectively. Although both methods are commonly used in gene expression analysis, they provide complementary information. GSEA focuses on identifying differentially expressed gene sets, while GSVA provides a more continuous measure of pathway activity. By using both methods, we aim to gain a more complete understanding of the underlying biology of PE.

### Construction of a diagnostic model for preeclampsia

To obtain the PE diagnostic models of the combined datasets, we screened a logistic regression analysis based on NRDEGs by *p*-value < 0.05, a logistic regression model was constructed, and then the molecular expression of NRDEGs included in the model was displayed as a forest plot. Then, the NRDEGs included in the logistic regression model were subjected to the least absolute shrinkage and selection operator (LASSO) logistic regression analysis using the R package "glmnet" [31], aiming to avoid overfitting and improve generalization ability. The outcomes of the LASSO regression analysis were displayed using diagnostic model maps and variable trajectory maps.

The nomogram [32] displays the functional relationship between multiple independent variables by a cluster of disjoint line segments in a rectangular coordinate system. The nomogram was utilized to perform the interrelationships of NRDEGs included in the LASSO regression model using the R package "rms". Finally, based on the LASSO regression model, we assessed the precision and discrimination of the PE diagnostic model using the calibration curve and decision curve analysis (DCA) [33].

### Construction of necroptosis score and weighted gene association network analysis

Single-sample gene-set enrichment analysis (ssGSEA) could calculate the relative abundance of each gene in a dataset sample. Necroptosis score (N Score) is a measure of the overall expression of genes associated with NRDEGs, as determined by the ssGSEA algorithm. N Score was calculated for all samples based on the expression matrix of the combined datasets via the ssGSEA algorithm by the GSVA package. Finally, the N score's accuracy was evaluated by drawing a group comparison map using the R package "ggplot2" and the ROC curve using the R package "pROC."

Weighted Correlation Network Analysis (WGCNA) [34] is a systems biology technique used to describe the pattern of gene connection between several samples, which be used to find gene sets with highly synergistic alterations. We build a weighted co-expression network of potential NRDEGs by the R package "WGCNA" [35]. Subsequently, the variance of all the genes in the combined datasets was calculated, and hierarchical clustering trees were built based on the correlation coefficients between genes. The various cluster tree branches correspond to various gene modules. Various colors represent different modules. Then the module significance is determined by screening the top 25% of variant genes, establishing a minimum of 25 genes module, choosing an optimal soft threshold of 6, measuring a scale-free fitting index of 0.90, determining a shear height of 0.20, calculating the correlation between the N Score and each module's recording genes. Finally, the modules of |*r* value|> 0.30 were screened, and all modules' genes intersected with NRDEGs, respectively. All intersection genes obtained from different modules were key module genes. Only modules with intersection genes were retained and drawn by the Venn diagram.

### Protein–protein interaction network and analysis of key modules genes

Protein–protein interaction (PPI) network is composed of proteins interacting. The STRING database [36](https://string-db.org/) is a database that searches for connections between known and forecasted proteins. In this study, based on key module genes, a PPI network connected to differentially expressed genes was built using the STRING database (minimum needed interaction score: low confidence (0.150) as the standard) and visualized by Cytoscape [37] software.

To assess the diagnostic effect of key module gene's expression in PE. The receiver operating characteristic (ROC) curve [38] analyzes coordinate schema that can choose the best model, eliminate the second-best model, or establish the best threshold within the same model. The ROC curve is a composite measure of sensitivity and specificity for the continuous variables and the correlation between the two is demonstrated by the composition technique.

The ROC curves of key module genes in PE were plotted using the R package "pROC" and the AUC of the ROC curve was computed. The ROC curve's AUC typically ranges from 0.5 to 1. The diagnostic performance is better the closer the AUC is to 1. The accuracy was low when AUC was between 0.5 and 0.7, moderate when AUC was between 0.7 and 0.9, and high when AUC was above 0.9. Finally, the R package "igraph" was used to create a correlation presentation of chord diagrams based on Spearman's correlation analysis of key module genes. The absolute r value below 0.3 was weak, or no correlation, 0.3 to 0.5 was little correlation, 0.5 to 0.8 was moderate, and above 0.8 was a strong correlation.

### Construction of disease subtypes in preeclampsia

Consensus Clustering is a resampling-based algorithm used to identify each member and their subgroup number and verify the rationality of the clustering. It is a process that involves several iterations across dataset subsamples and provides an indicator of cluster stability and parameter choices by subsampling to introduce sampling variability. To identify different disease subtypes of PE, we classified key module genes into various clusters by consensus clustering [39]using the R package "ConsensusClusterPlus" [40] (50 iterations, 80% resampling rate Pearson correlation). To assess the diagnostic efficacy of key module gene expression in different disease subtypes of PE, the R package "pROC" was applied to plot the Curve ROC of key module genes in different disease subtypes of PE and calculate the AUC of the ROC curve.

### Immune infiltration analyses

CIBERSORT [41] (https://cibersortx.stanford.edu/) is based on linear support vector regression to deconvolute the transcriptome expression matrix to estimate the composition and abundance of immune cells in mixed cells. We calculated the proportion of 22 immune cell types from combined datasets using the CIBERSORT algorithm to verify the relationship between key module genes and the immune microenvironment. We displayed correlation heat maps by the R package "pheatmap." The enrichment score was set to > 0.

In addition, the immune cell infiltrates matrix of PE subtypes was analyzed by ssGSEA [42] with the R package "GSVA" based on the relative abundance of every immunocyte infiltrate in PE in each sample, filtering and outputting the samples with *p*-value < 0.05. Lastly, the correlation analysis results between key module genes and infiltrating immune cells in disease subtypes of PE were shown in correlation heat maps by the R package "pheatmap."

### Statistical analysis

All statistical charts and analyses were conducted using R (Version 4.2.0). Continuous variables were represented by the mean ± standard deviation (SD). A Wilcoxon rank-sum test was used to compare the two groups' differences. Utilize Spearman correlation analysis to calculate the correlation coefficient between various variables. The pROC package was also utilized to perform ROC analysis and the widely-used binary evaluation. A *p*-value < 0.05 was regarded as statistically significant in each test.

## Results

### Workflow chart

**Fig. 1:**
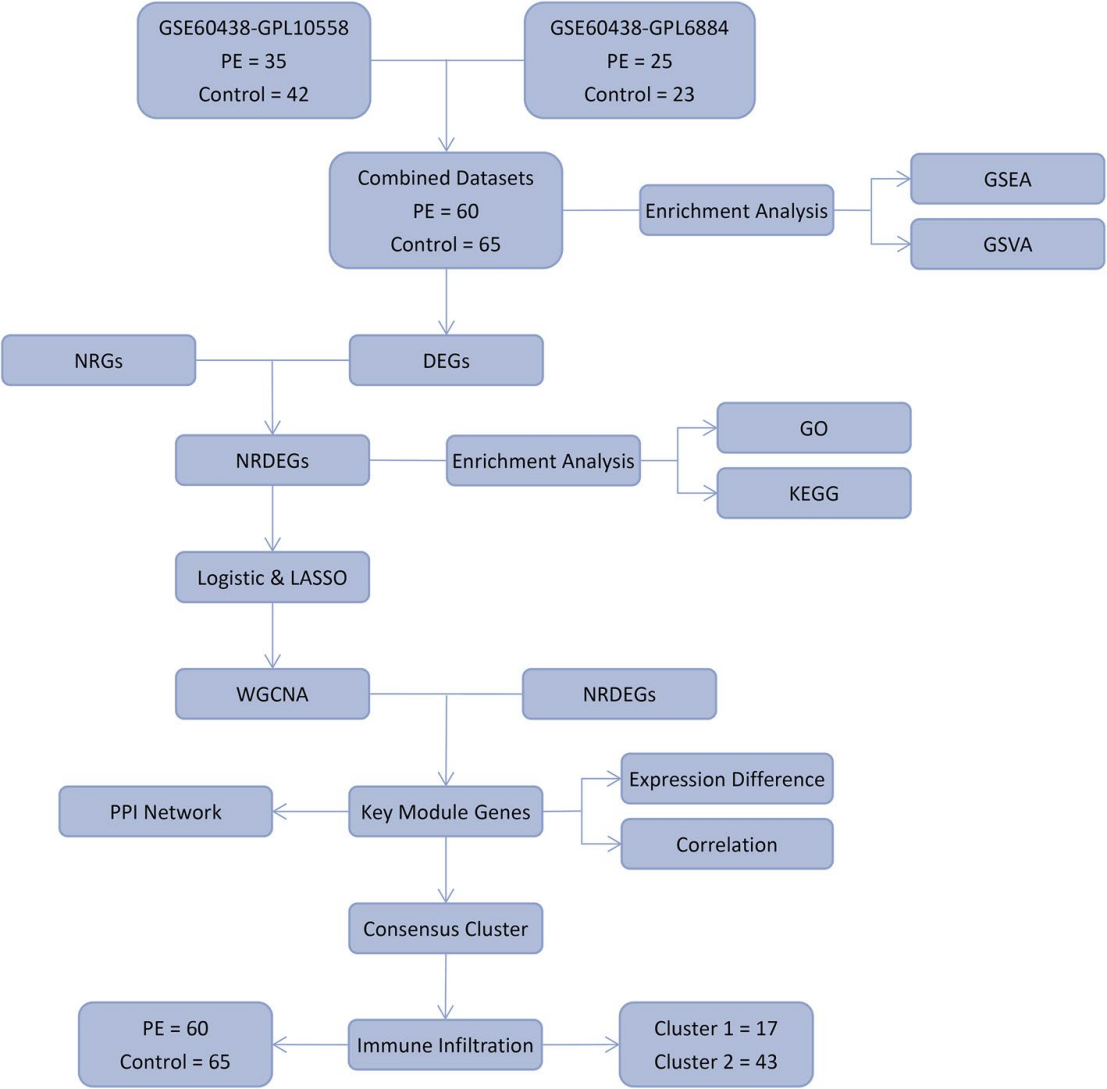
Flow chart for the comprehensive analysis of NRDEGs. PE, Pre-eclampsia; DEGs, Differentially Expressed Genes; NRGs, Necroptosis-Related Genes; NRDEGs, Necroptosis-Related Differentially Expressed Genes; GO, Gene Ontology; KEGG, Kyoto Encyclopedia of Genes and Genomes; GSEA, Gene Set Enrichment Analysis; GSVA, Gene Set Variation Analysis; LASSO, Least Absolute Shrinkage and Selection Operator; WGCNA, Weighted Correlation Network Analysis; PPI, Protein–Protein Interaction

To clarify the research process, we show our study's workflow in Fig. 1.

### Merging of preeclampsia datasets

**Fig. 2:**
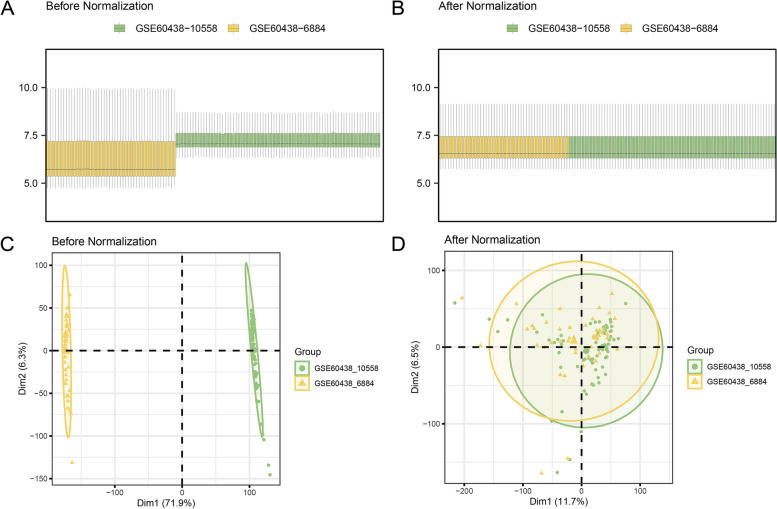
Batch effects removal of GSE60438. **A-B**. Combined datasets distribution boxplots before (**A**) and after (**B**) debatching. **C-D**. Combined datasets PCA plot before (**A**) and after (**B**) debatching. PCA, Principal Component Analysis; PE, Pre-eclampsia. Yellow is the dataset GSE60438-GPL6884; green is the dataset GSE60438-GPL10558

The batch effects in dataset GSE60438 were eliminated using the R package "sva" to obtain combined datasets. The distribution boxplot and PCA plot findings demonstrated that batch removal reduced the batch effect of samples in the PE dataset (shown in Fig. 2A-D).

### Identification of NRDEGs

**Fig. 3:**
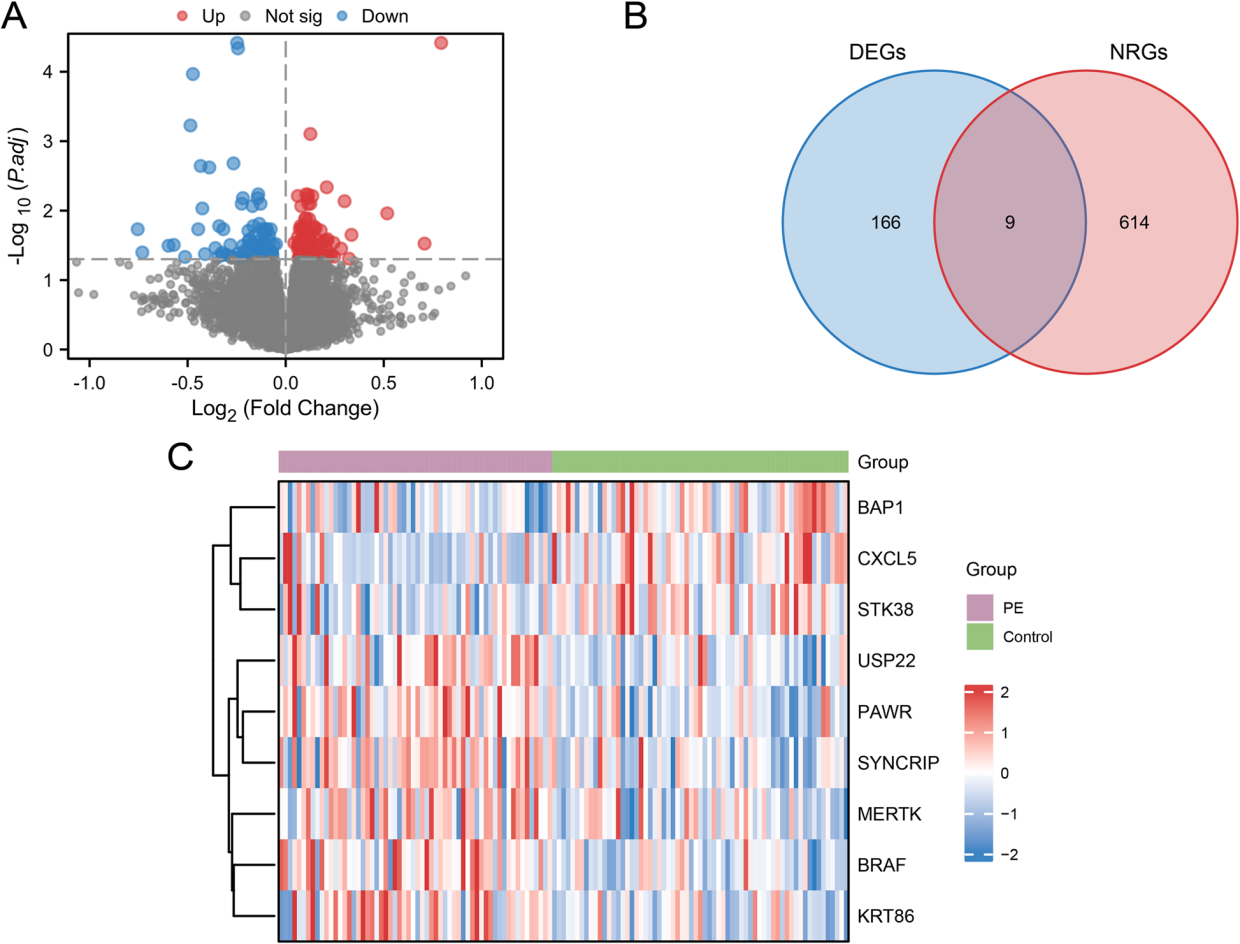
Combined datasets differential gene expression analysis. **A**. The volcano plot of DEGs of PE and control groups in combined datasets. **B**. Venn diagram displaying the overlap of genes between NRGs and combined datasets' DEGs. **C**. Clustered heatmap of NRDEGs in combined datasets. PE, Pre-eclampsia; DEGs, Differentially Expressed Genes; NRGs, Necroptosis-Related Genes; NRDEGs, Necroptosis-Related Differentially Expressed Genes. Green represents the control group; purple represents the PE group

The combined datasets' data were classified into PE and control groups. To explore the gene expression variations between the PE and control groups in combined datasets, we subjected the combined datasets to a discrepant analysis using the R package "limma" to obtain the DEGs of the two datasets. A total of 175 DEGs meeting |logFC|> 0 and adjust *p*-value < 0.05 were obtained. Among them, 85 genes were up-regulated (logFC > 0 and adjust *p*-value < 0.05) and 90 genes were down-regulated ( logFC < 0 and adjust *p*-value < 0.05). The volcano map visualized the DEGs (Fig. 3A). Secondly, 9 NRDEGs were obtained by taking the intersection between all the obtained DEGs and NRGs and drawing them using by Venn diagram. The nine NRDEGs were BRAF, PAWR, USP22, SYNCRIP, KRT86, MERTK, BAP1, CXCL5 and STK38 (Fig. 3B). Finally, the expression differences of NRDEGs between different sample groups in combined datasets were analyzed, and heatmap of the analysis results was presented by the R package "pheatmap" (Fig. 3C).

### GO and KEGG pathway enrichment analysis of NRDEGs

**Fig. 4:**
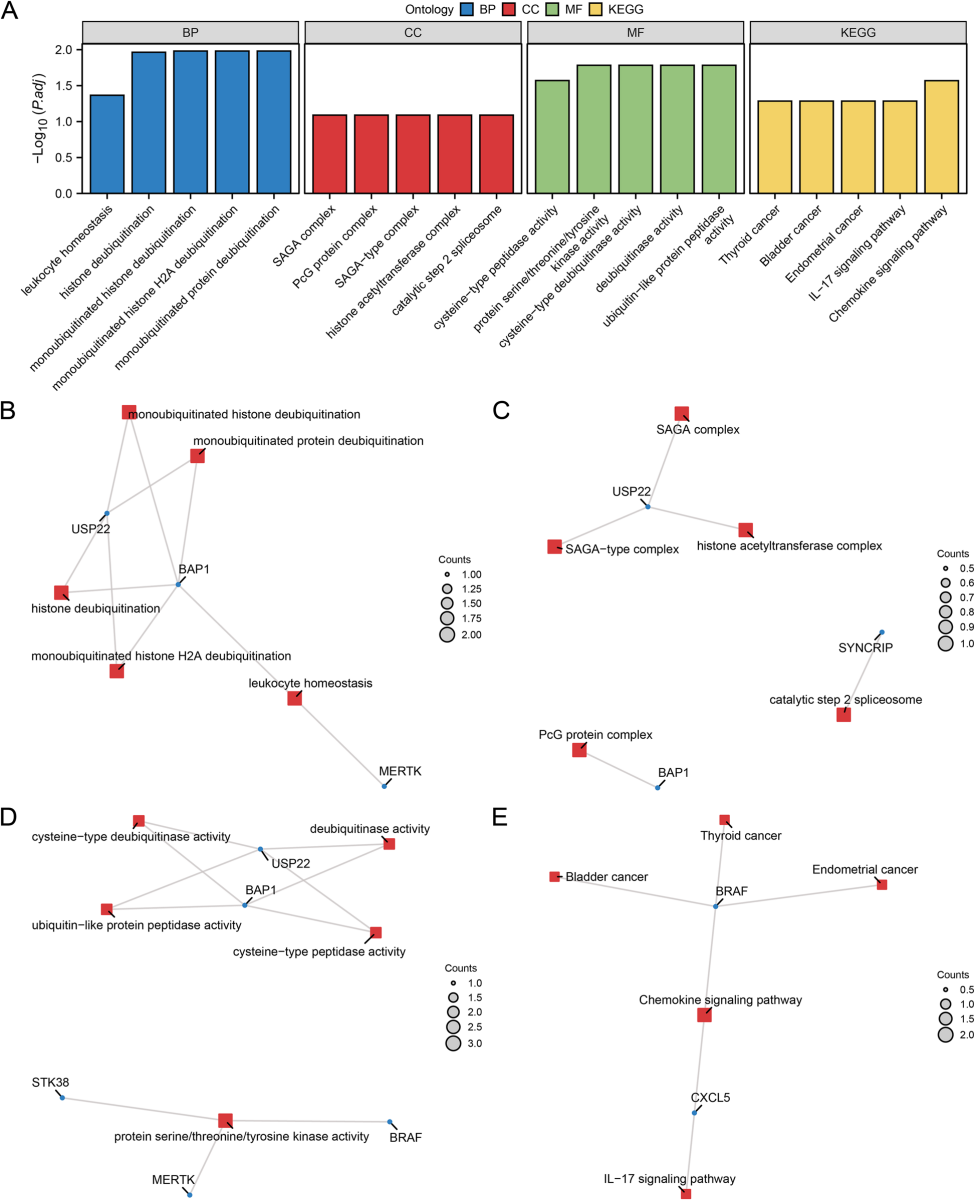
GO and KEGG enrichment analysis for NRDEGs. **A**. The GO and KEGG enrichment results of NRDEGs are shown in the histogram: biological processes (BP), cell components (CC), molecular functions (MF) and biological pathways (KEGG). The x-coordinate is GO terms and KEGG terms. **B-E**. The results of GO and KEGG enrichment analysis of NRDEGs are shown in the network diagram: BP (**B**), CC (**C**), MF (**D**) and KEGG (**E**). NRDEGs, Necroptosis-Related Differentially Expressed Genes; GO, Gene Ontology; KEGG, Kyoto Encyclopedia of Genes and Genomes; BP, Biological Process; CC, Cellular Component; MF, Molecular Function. Red nodes represent BP, CC, MF and KEGG entries; blue nodes represent molecules; lines represent relationships between entries and molecules. The GO and KEGG enrichment analysis screening criteria were adj. p-value < 0.05, FDR < 0.25 was considered statistically significant, and adj.p correction method Benjamini-Hochberg (BH)

Nine NRDEGs were analyzed by GO and KEGG pathway analysis (shown in Table 2). The functional enrichment of NRDEGs was examined using the three components of the GO annotations: CC, BP, and MF. The NRDEGs in PE were mainly related to BP (such as monoubiquitinated histone deubiquitination, monoubiquitinated histone H2A deubiquitination, monoubiquitinated protein deubiquitination, histone deubiquitination and leukocyte homeostasis); CC (such as SAGA complex, PCG protein complex, SAGA-type complex, histone acetyltransferase complex and catalytic step 2 spliceosome); MF (such as protein serine/threonine/tyrosine kinase activity, cysteine-type deubiquitinase activity, deubiquitinase activity, ubiquitin-like protein peptidase activity and cysteine-type peptidase activity). KEGG analysis was conducted to determine the relationship between NRDEGs and signaling pathways. The NRDEGs were mainly associated with a chemokine signaling pathway, thyroid cancer, bladder cancer, endometrial cancer and IL-17 signaling pathway. The findings of an investigation of KEGG and GO pathway enrichment were visualized by bar charts (Fig. 4A). Meanwhile, the network diagrams of BP, CC, MF and pathway were created using the results of GO and KEGG's enrichment analysis (Fig. 4B-E).

**Table 2:** Results of GO and KEGG enrichment analysis for NRDEGs

ONTOLOGY	ID	Description	GeneRatio	BgRatio	pvalue	p.adjust	qvalue
BP	GO:0035521	monoubiquitinated histone deubiquitination	2/9	29/18800	8.22E-05	1.04E-02	6.76E-03
BP	GO:0035522	monoubiquitinated histone H2A deubiquitination	2/9	29/18800	8.22E-05	1.04E-02	6.76E-03
BP	GO:0035520	monoubiquitinated protein deubiquitination	2/9	34/18800	1.13E-04	1.04E-02	6.76E-03
BP	GO:0016578	histone deubiquitination	2/9	40/18800	1.57E-04	1.09E-02	7.04E-03
BP	GO:0001776	leukocyte homeostasis	2/9	89/18800	7.81E-04	4.31E-02	2.79E-02
CC	GO:0000124	SAGA complex	1/9	21/19594	9.61E-03	8.14E-02	3.81E-02
CC	GO:0031519	PcG protein complex	1/9	34/19594	1.55E-02	8.14E-02	3.81E-02
CC	GO:0070461	SAGA-type complex	1/9	36/19594	1.64E-02	8.14E-02	3.81E-02
CC	GO:0000123	histone acetyltransferase complex	1/9	87/19594	3.93E-02	8.14E-02	3.81E-02
CC	GO:0071013	catalytic step 2 spliceosome	1/9	88/19594	3.97E-02	8.14E-02	3.81E-02
MF	GO:0004712	protein serine/threonine/tyrosine kinase activity	3/9	446/18410	1.06E-03	1.65E-02	5.37E-03
MF	GO:0004843	cysteine-type deubiquitinase activity	2/9	106/18410	1.15E-03	1.65E-02	5.37E-03
MF	GO:0101005	deubiquitinase activity	2/9	113/18410	1.31E-03	1.65E-02	5.37E-03
MF	GO:0019783	ubiquitin-like protein peptidase activity	2/9	124/18410	1.57E-03	1.65E-02	5.37E-03
MF	GO:0008234	cysteine-type peptidase activity	2/9	178/18410	3.20E-03	2.69E-02	8.76E-03
KEGG	hsa04062	Chemokine signaling pathway	2/2	192/8164	5.50E-04	2.70E-02	4.63E-03
KEGG	hsa05216	Thyroid cancer	1/2	37/8164	9.04E-03	5.19E-02	8.91E-03
KEGG	hsa05219	Bladder cancer	1/2	41/8164	1.00E-02	5.19E-02	8.91E-03
KEGG	hsa05213	Endometrial cancer	1/2	58/8164	1.42E-02	5.19E-02	8.91E-03
KEGG	hsa04657	IL-17 signaling pathway	1/2	94/8164	2.29E-02	5.19E-02	8.91E-03

*GO* Gene Ontology, *BP* Biological Process, *CC* Cellular Component, *MF* Molecular Function, *KEGG* Kyoto Encyclopedia of Genes and Genomes, *NRDEGs* Necroptosis-Related Differentially Expressed Genes

### Gene set enrichment analysis

**Fig. 5:**
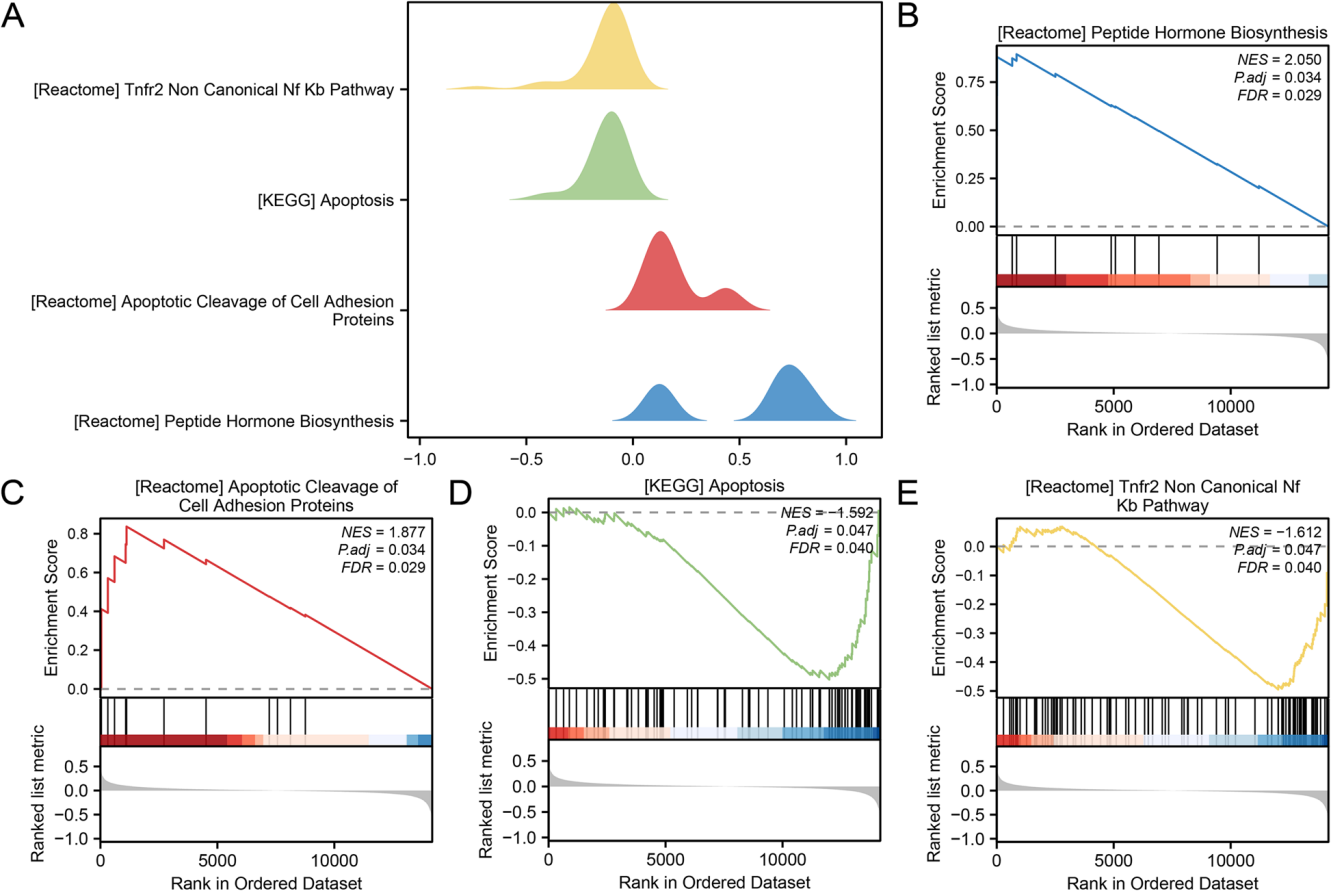
GSEA for combined datasets. **A** A total of 4 biological functions of GSEA in combined datasets were displayed by mountain map. **B-E**. The GSEA revealed PE-associated DEGs significantly enriched in peptide hormone biosynthesis (**B**), apoptotic cleavage of cell adhesion proteins (**C**), apoptosis (**D**) and TNFR2 non-canonical NF-κB pathway (**E**). PE, Pre-eclampsia; GSEA, Gene Set Enrichment Analysis. The screening criteria for GSEA were adj.*p*-value < 0.05 and FDR < 0.25

GSEA performed a relationship between the expression of all genes in combined datasets and BP, CCs, and MF. Twenty pathways were obtained by GSEA (Fig. 5A), and the specific results are shown in Table 3. According to GSEA, PE had considerably higher levels of gene expression related to peptide hormone synthesis (Fig. 5B), cell adhesion protein cleavage during death (Fig. 5C), apoptosis (Fig. 5D), and TNFR2 non-canonical NF-B pathway (Fig. 5E).

**Table 3:** Results of GSEA for combined datasets

ID	setSize	enrichmentScore	NES	pvalue	p.adjust	qvalues
REACTOME_HORMONE_LIGAND_BINDING_RECEPTORS	12	9.12E-01	2.06E + 00	1.94E-03	3.42E-02	2.92E-02
REACTOME_LAMININ_INTERACTIONS	30	7.45E-01	2.05E + 00	2.06E-03	3.42E-02	2.92E-02
REACTOME_PEPTIDE_HORMONE_BIOSYNTHESIS	13	8.95E-01	2.05E + 00	1.97E-03	3.42E-02	2.92E-02
REACTOME_NON_INTEGRIN_MEMBRANE_ECM_INTERACTIONS	59	6.48E-01	2.02E + 00	2.27E-03	3.60E-02	3.07E-02
REACTOME_METABOLISM_OF_STEROID_HORMONES	29	7.30E-01	1.99E + 00	2.07E-03	3.42E-02	2.92E-02
NABA_BASEMENT_MEMBRANES	36	6.92E-01	1.98E + 00	2.13E-03	3.42E-02	2.92E-02
KEGG_STEROID_HORMONE_BIOSYNTHESIS	54	6.42E-01	1.96E + 00	2.26E-03	3.60E-02	3.07E-02
WP_PRADERWILLI_AND_ANGELMAN_SYNDROME	62	6.22E-01	1.94E + 00	2.31E-03	3.61E-02	3.09E-02
REACTOME_SMOOTH_MUSCLE_CONTRACTION	36	6.73E-01	1.93E + 00	2.13E-03	3.42E-02	2.92E-02
REACTOME_MET_ACTIVATES_PTK2_SIGNALING	30	6.97E-01	1.92E + 00	2.06E-03	3.42E-02	2.92E-02
WP_GLUCURONIDATION	25	7.18E-01	1.90E + 00	2.05E-03	3.42E-02	2.92E-02
WP_H19_ACTION_RBE2F1_SIGNALING_AND_CDKBETACATENIN_ACTIVITY	13	8.24E-01	1.89E + 00	1.97E-03	3.42E-02	2.92E-02
REACTOME_CELL_JUNCTION_ORGANIZATION	80	5.73E-01	1.89E + 00	2.40E-03	3.70E-02	3.16E-02
REACTOME_TIGHT_JUNCTION_INTERACTIONS	28	6.93E-01	1.89E + 00	2.09E-03	3.42E-02	2.92E-02
REACTOME_APOPTOTIC_CLEAVAGE_OF_CELL_ADHESION_PROTEINS	11	8.37E-01	1.88E + 00	1.88E-03	3.42E-02	2.92E-02
REACTOME_CELL_CELL_JUNCTION_ORGANIZATION	55	6.06E-01	1.86E + 00	2.28E-03	3.60E-02	3.07E-02
REACTOME_ATTACHMENT_AND_ENTRY	18	7.50E-01	1.85E + 00	2.02E-03	3.42E-02	2.92E-02
REACTOME_ANDROGEN_BIOSYNTHESIS	10	8.48E-01	1.84E + 00	1.88E-03	3.42E-02	2.92E-02
KEGG_APOPTOSIS	81	-5.02E-01	-1.59E + 00	3.41E-03	4.66E-02	3.98E-02
REACTOME_TNFR2_NON_CANONICAL_NF_KB_PATHWAY	95	-4.95E-01	-1.61E + 00	3.42E-03	4.66E-02	3.98E-02

*GSEA* Gene Set Enrichment Analysis

### Gene set variation analysis

To explore biological differences between PE and control groups, we performed GSVA and screened out thirty-three statistically significant pathways (shown in Table S2). These pathways were Mitochondrial fatty acid beat oxidation of saturated fatty acids; Glycolysis; Hypoxia; Activated NTRK2 signals through PI3K; AKT pathway targets; Mitochondrial long chain fatty acid beta-oxidation; TP53 regulates transcription of death receptors and ligands; Beta oxidation of decanoyl CoA to octanoyl CoA; Erythropoietin activates STAT5; Interleukin-1 processing; Adipogenic genes repressed by SIRT1; TRIF mediated programmed cell death; IFN response not via IRF3; MET activates PI3k-AKT signaling; IFN-a pathway, etc. Then, visualized by boxplot (Fig. S1A) and heatmap (Fig. S2B) (criteria for screening pathway:| logFC |> 0.25 and adjust *p*-value < 0.05).

### Construction of a diagnostic model for preeclampsia

**Fig. 6:**
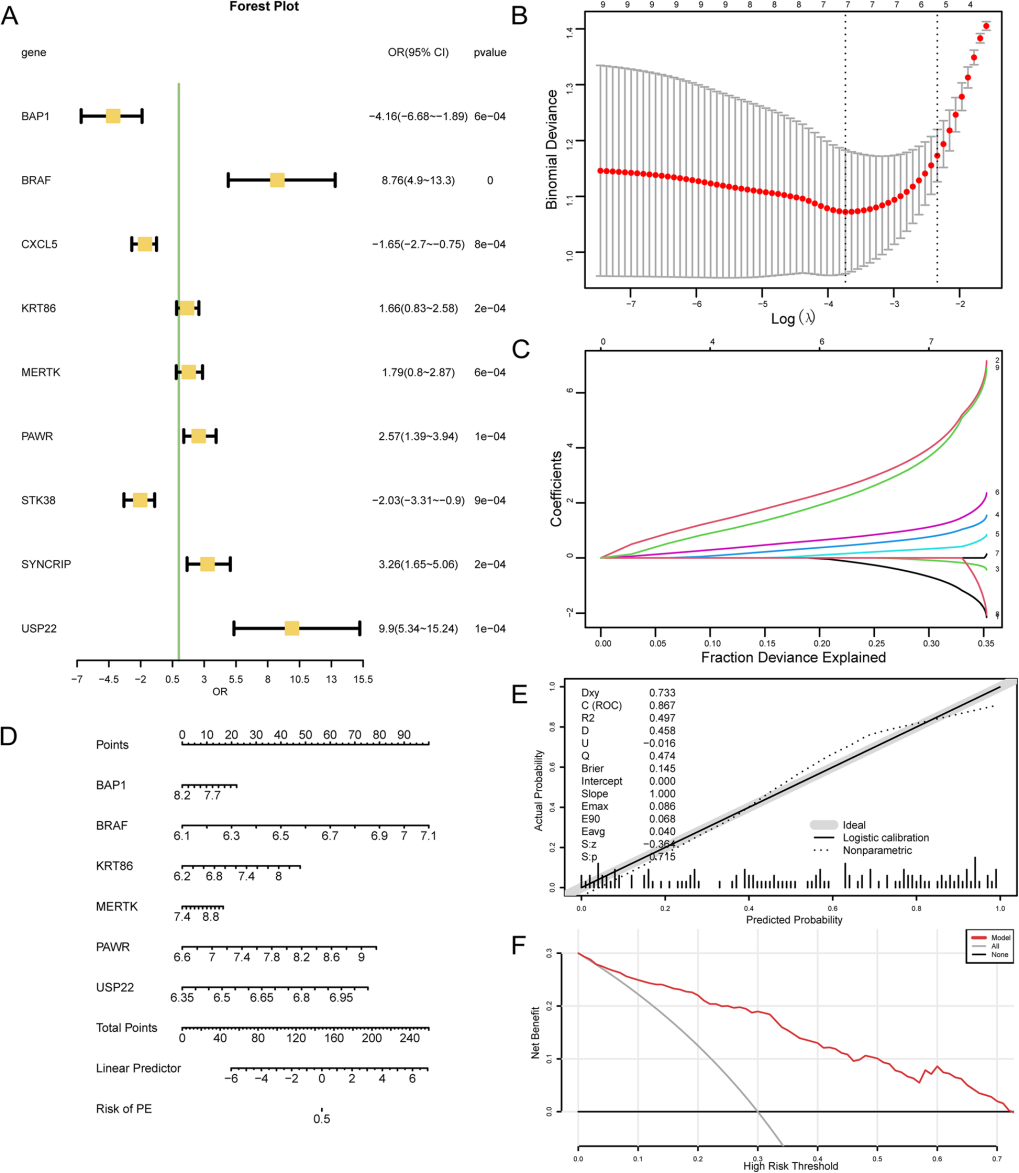
Diagnostic model of PE. **A**. Forest plot of a logistic regression model. The first column is the variable name, six NRDEGs invalid lines: one perpendicular to the X-axis, usually X = 1 or 0. The line segment is the analysis result of each included study, namely 95%CI. The shorter the line segment, the smaller 95%CI, the more accurate the result, and the greater the weight. The data corresponding to OR (95%CI) in the third column is the effective value and 95%CI of the aggregate results of each included study and meta-analysis. The fourth column was *p*-value, *p*-value < 0.5, indicating statistical significance. **B-C**. Diagnostic model plots (**B**) and variable trajectories plots (**C**) of the LASSO regression model. **D**. Nomogram of 6 NRDEGs in LASSO regression model. Names of variables in the prediction model: 6 NRDEGs on the far left. Score: includes the score of a single item (Point in the figure), which represents the score of a single item corresponding to different values of each variable, and the Total score (Total Point), which represents the total score of a single item related to the value of all variables. Linear Predictor: Linear predictive value. Risk of PE: indicates the risk value probability of PE. **E–F**. A calibration curve (**E**) and DCA (**F**) of 6 NRDEGs in the PE diagnostic model. The ordinate is the net benefit, and the abscissa is the threshold probability. OR, odds ratio; PE, Pre-eclampsia; NRDEGs, Necroptosis-Related Differentially Expressed Genes; LASSO, Least Absolute Shrinkage and Selection Operator; DCA, Decision Curve Analysis

To establish the diagnostic worth of nine NRDEGs in PE, we built a logistic regression model based on the nine NRDEGs, including BRAF, PAWR, USP22, SYNCRIP, KRT86, MERTK, BAP1, CXCL5 and STK38 of logistic regression, and visualized by forest plot (Fig. 6A). Then, based on the 9 NRDEGs, the LASSO regression model was constructed and visualized by LASSO regression model plot (Fig. 6B) and LASSO variable trajectory plot (Fig. 6C). The results showed that the LASSO regression model including 6 NRDEGs, such as BAP1, BRAF, KRT86, MERTK, PAWR, and USP22. The 6 NRDEGs were used to construct a nomogram for interrelationships of NRDEGs (Fig. 6D). BRAF and PAWR had high diagnostic utility for PE, while MERTK had low diagnostic utility. In addition, to judge the accuracy and resolution of the diagnostic model for PE, a calibration curve was drawn with calibration analysis, and the predictive effect of the model on the actual results was evaluated according to the matching between the actual probabilities and the predicted probabilities of the model under different conditions (Fig. 6E). Calibration curve of PE diagnostic model shows that the calibration line shown by the dotted line is slightly different from the diagonal line of the ideal model, but it is close to matching. Further, DCA assessed the role of the PE diagnostic model in clinical utility, and results were demonstrated (Fig. 6F). When the line of the model is higher than all positive and all negative in a particular range, the larger the range, the more net income, and the better the model effect. The results show that the line stability of the model is higher than all positive and all negative in a certain range, the net income of the model is more, and the effect of the model is better.

### Construction of necroptosis score and weighted gene association network analysis

**Fig.7:**
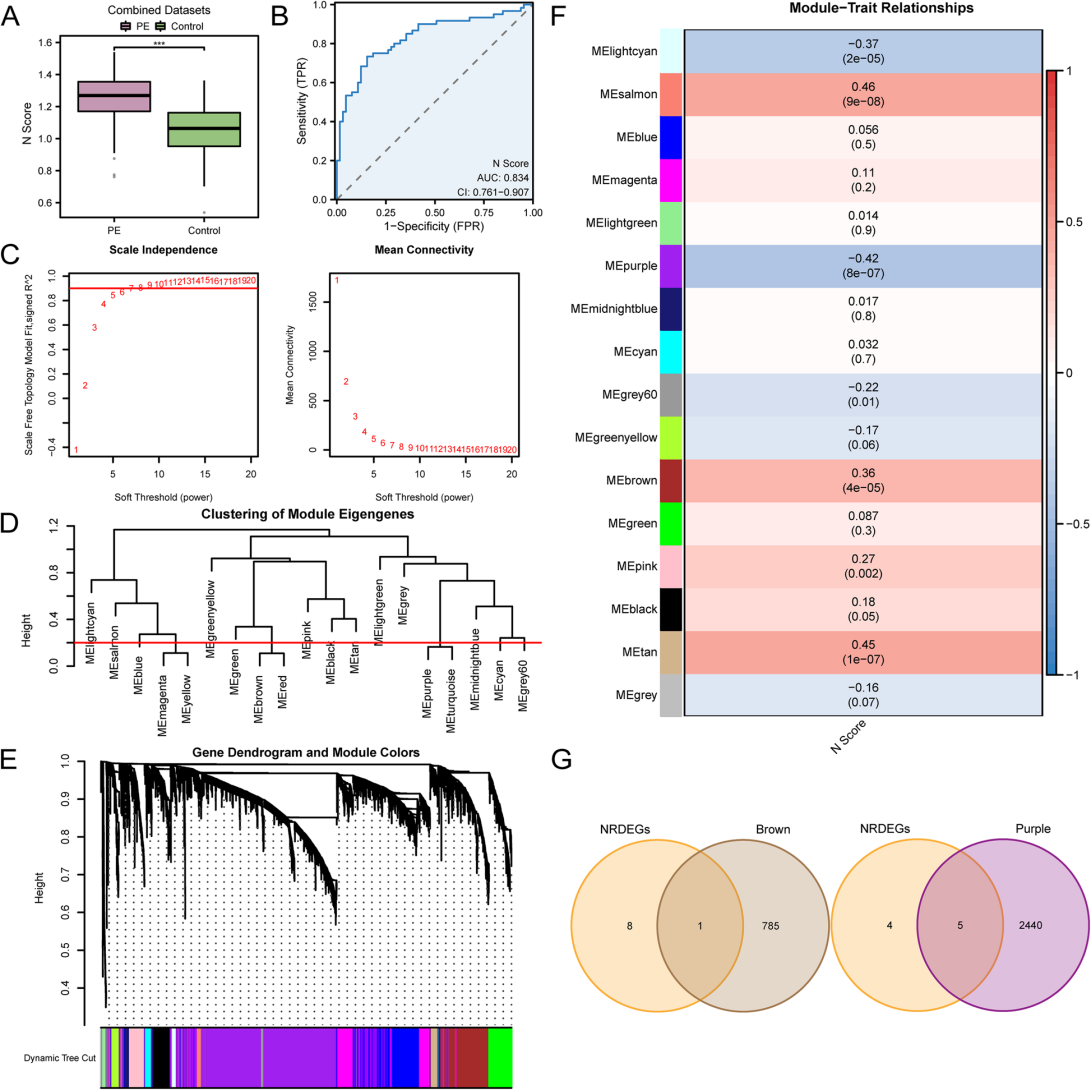
WGCNA for combined datasets. **A-B**. Results of N Score between PE and control groups in combined datasets by group comparison boxplot (**A**) and ROC curve (**B**). **C**. Scale-free network display of the optimal soft threshold in WGCNA. The left graph displays the optimal soft threshold, while the right graph displays network connectivity with various soft thresholds. **D**. Module clustering results of genes with the top 25% variance. **E**. Clustering results for genes with the top 25% variance, the upper part was hierarchical clustering dendrogram, and the lower part was gene modules. **F**. Correlation analysis between cluster modules of genes with top 25% variation and N score. **G**. Venn diagram of 9 NRDEGs with MEbrown, MEpurple modules. N Score, Necroptosis Score; PE, Pre-eclampsia; ROC, Receiver Operating Characteristic; WGCNA, Weighted Gene Co-Expression Network Analysis; NRDEGs, Necroptosis-Related Differentially Expressed Genes. ***: *p*-value < 0.001. AUC has a certain accuracy at 0.7–0.9. Red represents the PE group; blue represents the control group

The N Score of all samples was calculated using the ssGSEA method based on the expression of six NRDEGs in the diagnostic model. The difference in N Score was highly statistically significant between PE and the control group (*p*-value < 0.001) and visualized by boxplots (Fig. 7A). We utilized a ROC curve to illustrate the clinical usefulness of N Score in PE diagnosis to investigate its advantages. As shown in Fig. 7B, the AUC value of the N Score was 0.834 (CI = 0.761–0.907), which is considered capable of diagnosing PE with excellent specificity and sensitivity.

Based on PE-related combined datasets, WGCNA was used for genes with the highest 25% variance in combined datasets to identify the co-expression module. As shown in Fig. 7C, the scale-free fitting index is on the vertical axis, while the soft threshold is on the horizontal axis. The network is more consistent with scale-free network features the higher the scale-free fitting index is. The outcome demonstrated that the ideal soft threshold, based on the scale-free network, was six when the scale-free fitting index was 0.90. And then, based on the co-expression network with the optimal soft threshold, 16 modules, including MElightcyan, MEsalmon, MEblue, MEmagenta, MElightgreen, MEpurple, MEmidnightblue, MEcyan, MEgrey, MEgreenyellow, MEbrown, MEgreen, MEpink, MEblack, MEtan and MEgrey were identified by clustering tree (Fig. 7D) and visualized by hierarchical clustering (Fig. 7E).

Finally, the correlation between 16 modular genes and the N-score in the combined datasets was visualized (Fig. 7F). |*r* value|> 0.30 as the standard, five modules such as MElightcyan value (|*r* = 0.37|), MEsalmon value (|*r* = 0.46 |), MEpurple value (|*r* = 0.43|), MEbrown value (|*r* = 0.36 |) and MEtan value (|*r* = 0.45 |) were screened. A total of 9 NRDEGs were intersected with the genes included in the previous five modules, and only the modules with intersection genes were plotted as Venn diagrams (Fig. 7G). A total of 6 key module genes were obtained, namely PAWR, SYNCRIP, MERTK, CXCL5, STK38 and KRT86. Interestingly, these three key module genes (PAWR, MERTK, KRT86) are part of the model that calculates the N score.

### Construction of protein–protein interaction network and analysis of key module genes

**Fig. 8:**
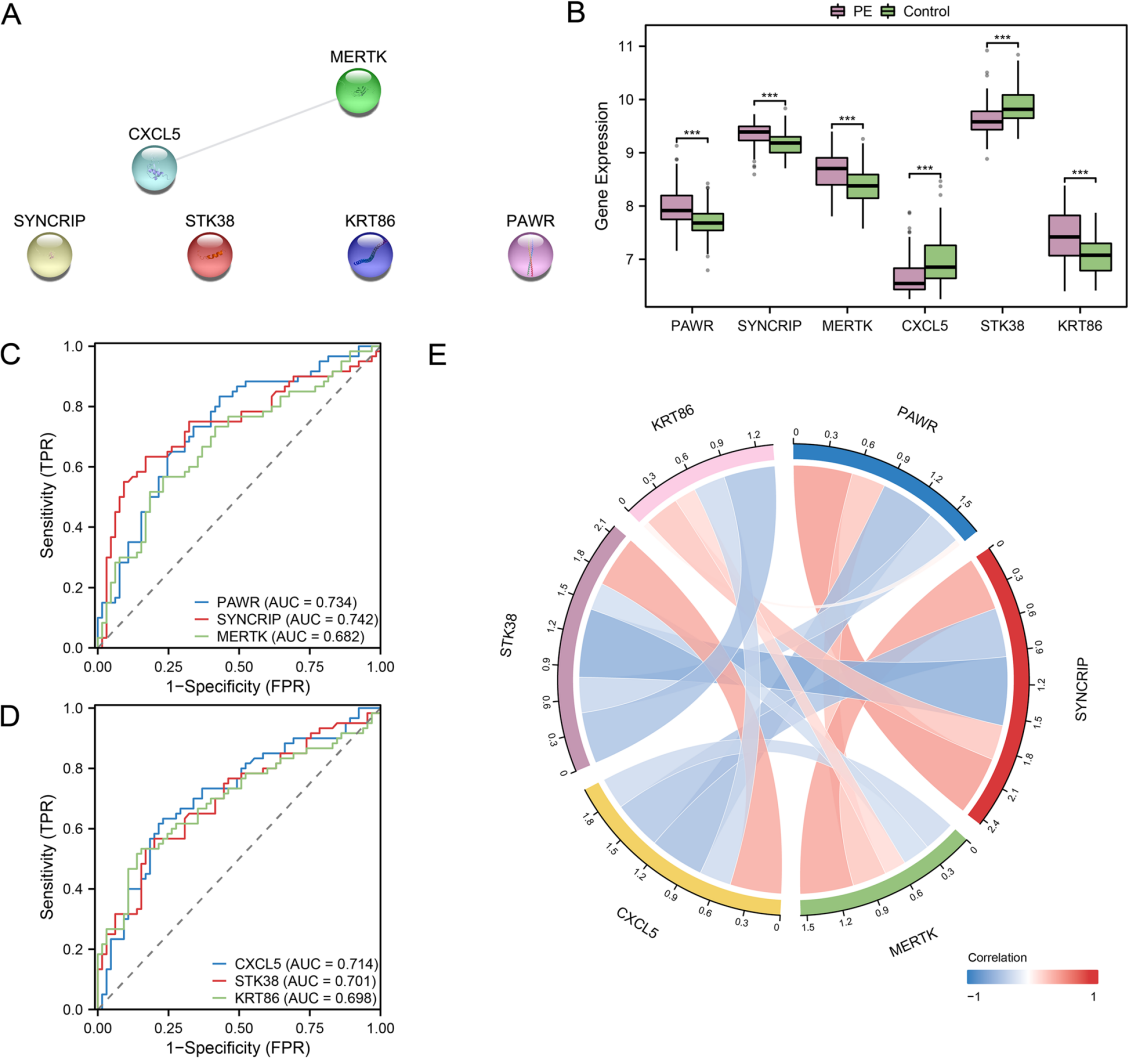
PPI network, expression difference and correlation analysis. **A**. PPI network of key module genes. **B-E**. Group comparison boxplot (**B**), ROC curve (**C-D**), and correlation circle plot (**E**) of key module genes in combined datasets. PPI Network, Protein–protein Interaction Network; ROC, Receiver Operating Characteristic; PE, Pre-eclampsia. ***: *p*-value < 0.001. Pink represents the PE group, green represents the control group. AUC has low accuracy at 0.5–0.7 and certain accuracy at 0.7–0.9. Red denotes a positive correlation, whereas blue denotes a negative correlation. The connecting string represents the correlation between genes, and the wider the band and the darker the color, the larger the absolute value of the correlation coefficient. When the absolute *r* value is between 0.5 and 0.8, it is moderately correlated

The STRING database was used for the PPI analysis of six key module genes, and Cytoscape V3.9.0 was used for visualization (Fig. 8A). The key module genes (CXCL5 and MERTK) were selected from the PPI network with interaction threshold scores ≥ 0.15. PAWR, SYNCRIP, MERTK, CXCL5, STK38 and KRT86 were highly statistically significant (*p*-value < 0.001) between PE and control groups in combined datasets by grouping comparison box plot (Fig. 8B). Then, the ROC curve results of the six key module genes in combined datasets were shown in Fig. 8C-D, which showed that PAWR, SYNCRIP, CXCL5 and STK38 showed a certain accuracy (0.7 < AUC < 0.9); MERTK and KRT86 showed low accuracy (0.5 < AUC < 0.7). Finally, the correlation circle diagram (Fig. 8E) was drawn based on the correlation of the entire expression matrix of the six key module genes, which showed the strongest positive correlations between PAWR and SYNCRIP and the strongest negative correlations between SYNCRIP and STK38.

### Immune infiltration analysis of the preeclampsia dataset

**Fig.9:**
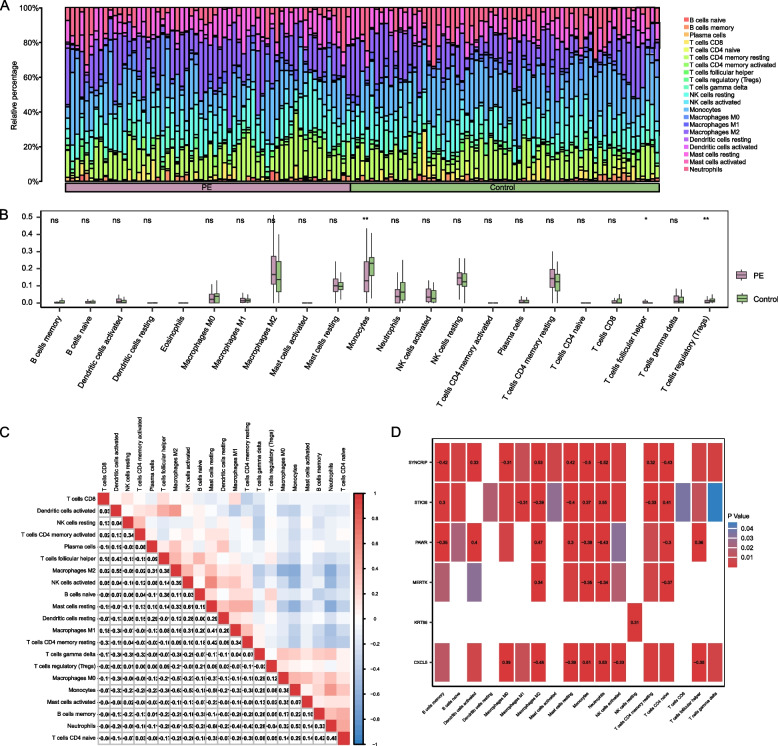
Combined datasets immune infiltration analysis by CIBERSORT algorithm. **A**. Proportion bar chart of immune cells infiltration analysis in combined datasets. **B**. Group comparison chart of Immune cells infiltration analysis in combined datasets. **C**. Correlation heatmap of immunocyte infiltration abundance for PE and control groups in combined datasets. **D**. Correlation heatmap between key module genes and immunocyte infiltration abundance for PE and control groups in combined datasets. PE, Pre-eclampsia. Green represents the control group; pink represents the PE group

The CIBERSORT algorithm analyzed the 22 categories of immune cells. The proportion bar chart revealed that macrophages M2, monocytes, T cells follicular helper, NK cells resting and T cells CD4 memory resting showed a higher abundance of infiltration than other immune cells (Fig. 9A). Compared with the control group, the group comparison chart demonstrated that the infiltration abundance of monocytes, T cells follicular helper and T cells regulatory displayed a statistically significant decrease (Fig. 9B). The correlation heatmap showed that more immune cells correlate negatively with PE than a positive correlation (Fig. 9C). Using correlation heatmaps, we investigated the relationship between immune cell infiltration abundance and key module genes in the PE datasets (Fig. 9D). According to the findings. neutrophils and monocytes showed a moderate positive correlation with CXC15 (*r*-value = 0.53, *r* value = 0.51) and displayed a moderate negative correlation with SYNCRIP (*r*-value = -0.52, *r* value = -0.50). In contrast, macrophages M2 showed a positive correlation with SYNCRIP (*r*-value = 0.53) and displayed a negative correlation with CXC15 (*r*-value = -0.48).

### Construction of preeclampsia-related disease subtypes

**Fig. 10:**
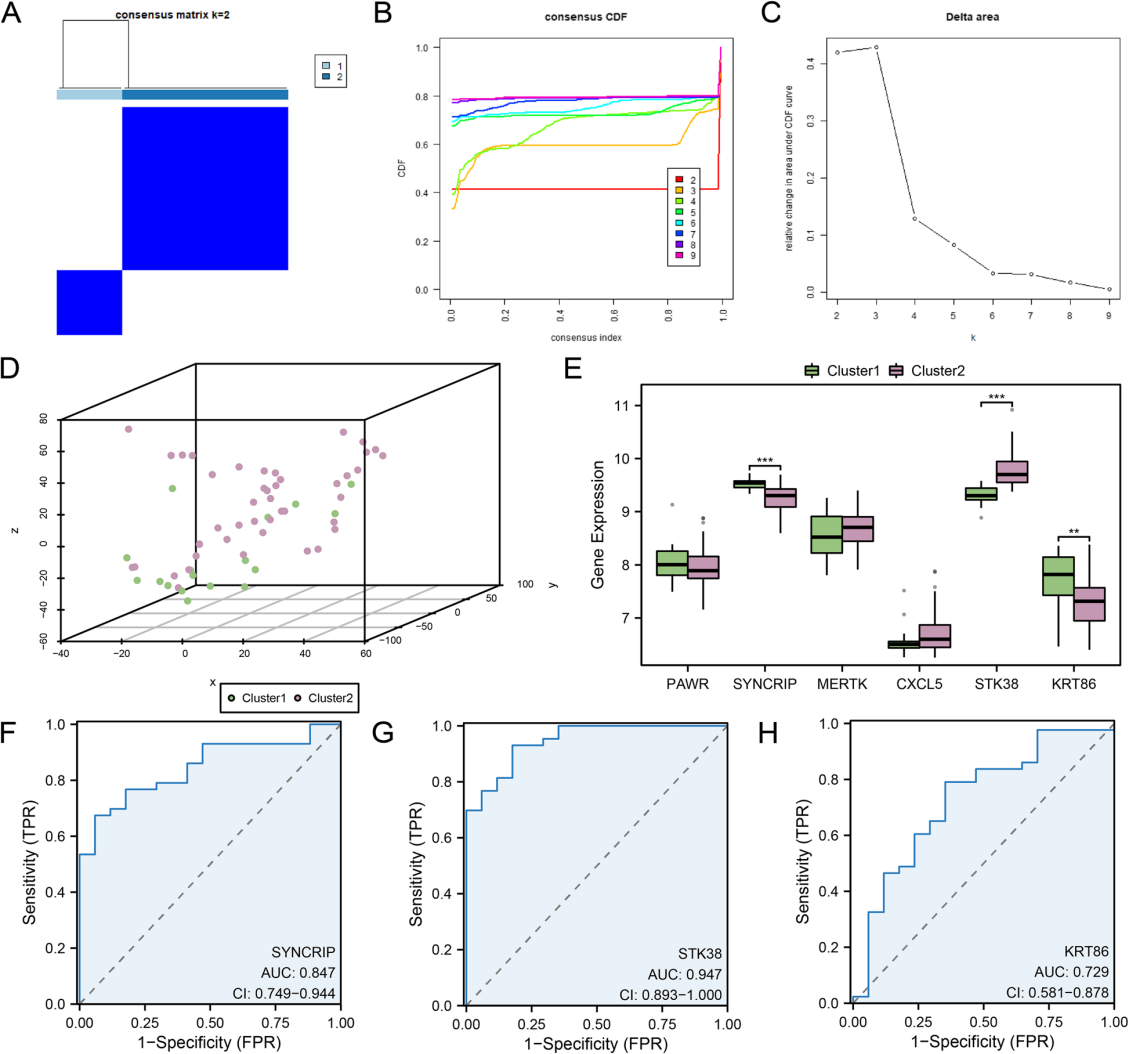
Consensus clustering analysis for hub genes. **A**. Consistent clustering results in different disease subtypes of PE. **B-C**. CDF plot (**B**) and Delta plot (**C**) in two disease subtypes of PE by consistency cluster analysis. **D**. 3D PCA map of two disease subtypes of PE. **E–H**. Group comparison boxplots (**E**) and ROC curves (**F–H**) of key module genes in PE disease subtypes. PE, Pre-eclampsia; CDF, Empirical Cumulative Distribution Function; PCA, Principal Component Analysis. Green represents Cluster1; pink represents Cluster2. **: *p*-value < 0.01; ***: *p*-value < 0.001. AUC has a certain accuracy between 0.7 and 0.9, while AUC has a high accuracy above 0.9

We investigated key module gene expression variations in PE samples using combined datasets. Based on six key module genes' expression, we identified two preeclampsia-related disease subtypes by consensus clustering analysis using the R package "ConsensusClusterPlus" with cluster 1 containing 17 samples and cluster 2 containing 43 samples. (Fig. 10A-C). Two subtypes showed significant differences for three-dimensional PCA (Fig. 10D).

To further verify the expression differences of key module genes in PE disease subtypes, the connection and difference between the key module genes' expression levels and the two PE subtypes were investigated by boxplot(Fig. 10E). The boxplot results showed three key module genes were statistically significant, including SYNCRIP and STK38 (*p*-value < 0.001) and KRT86 (*p*-value < 0.01). Finally, to investigate the clinical benefits of the three key module genes, we employed a ROC curve to investigate those values on differentiating PE subtypes (Fig. 10F-H). ROC curve showed that STK38 showed high accuracy (AUC > 0.9); SYNCRIP and KRT86 showed a sure accuracy (0.7 < AUC < 0.9).

### Analysis of immune infiltration in preeclampsia disease subtypes

**Fig. 11:**
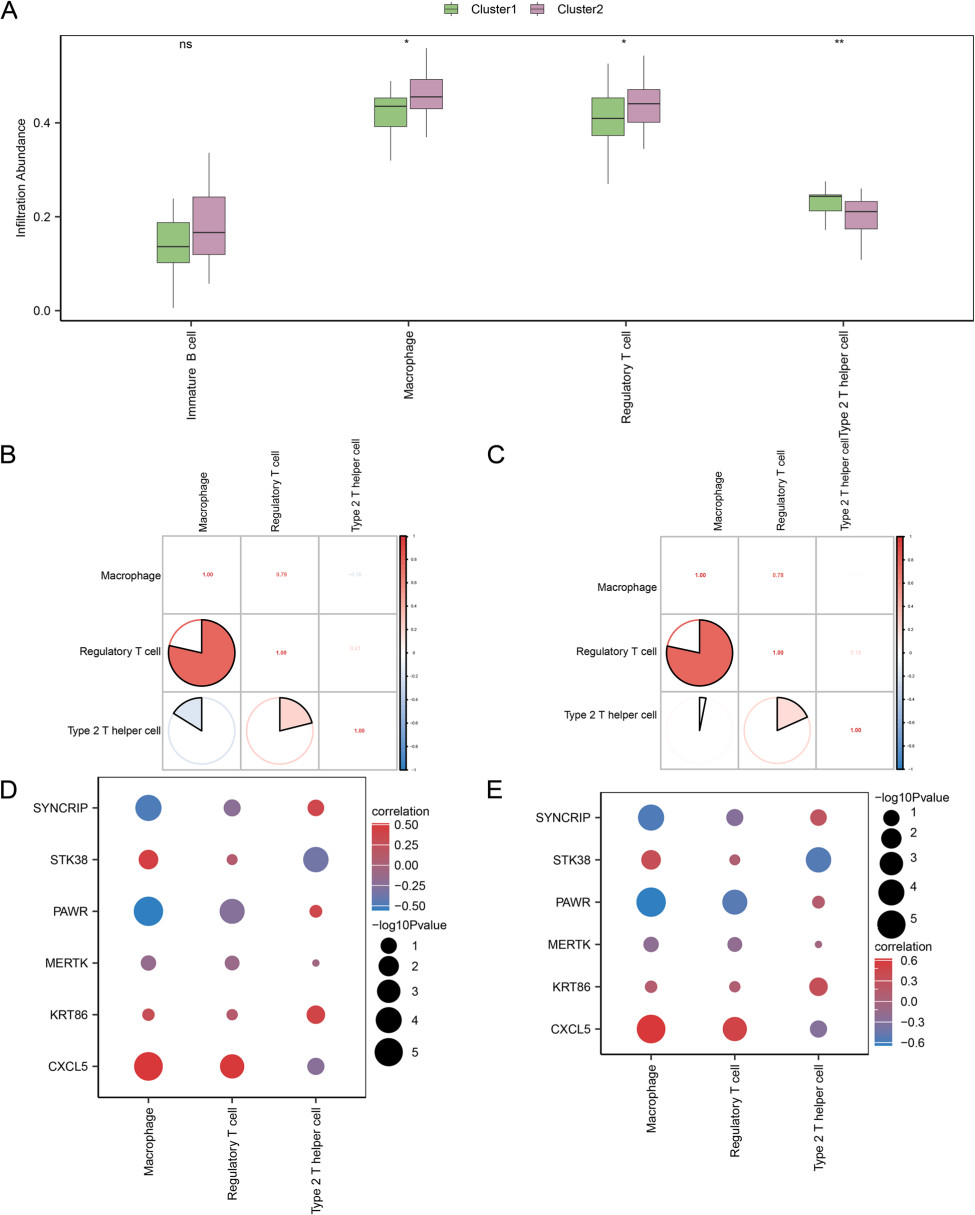
Consensus clustering immune infiltration analysis by ssGSEA Algorithm. **A**. Group comparison boxplot of immune cells in PE disease subtypes. **B-C**. Correlation analysis's results about immune cell infiltrate abundance in PE subtypes A (**B**) and B (**C**). **D-E**. Heatmap of the correlation between immune cell infiltration abundance and key module genes in PE subtypes A (**D**) and B (**E**). ssGSEA, single-sample Gene-Set Enrichment Analysis; PE, Pre-eclampsia. Green represents Cluster1; pink represents Cluster2. *: *p*-value < 0.05; **: *p*-value < 0.01. Blue denotes a negative correlation, whereas red denotes a positive correlation

Based on the PE groups sample's expression matrix in combined datasets, the abundance of 28 immunological cells' infiltration in PE disease subtypes was calculated by the ssGSEA algorithm. Firstly, infiltrating abundance of immune cells with screening *p*-value < 0.05 in different subtypes were shown by the group comparison boxplot. The results revealed that the three immune cells had significant differences: among them, infiltration abundance of Type 2 T helper cells was highly statistically significant among different subtypes of preeclampsia (*p*-value < 0.01); macrophage and regulatory T cells were statistically significant (*p*-value < 0.05) (Fig. 11A).

Subsequently, the association of the three immune cells' infiltration abundance was analyzed in different PE subtypes by correlation heatmap. As shown in Fig. 11B-C, the correlation between macrophage and Type 2 T helper cells was different between in subtype A (Cluster1) and subtype B (Cluster2). Finally, the correlation between key modul e genes in the PE dataset and immunological cell infiltration abundance by correlation heatmap (Fig. 11D-E). The results demonstrated that subtypes A (Cluster1) and B (Cluster2) had similar correlations between immune cells and key module genes. Macrophages showed the strongest negative correlation with PAWR and the strongest positive correlation with CXCL5.

## Discussion

Preeclampsia is a severe complication of pregnancy and the primary cause of maternal mortality worldwide, causing significant economic and psychological impacts on families due to associated fetal growth restriction, preterm birth, and other complications. Spiral artery remodeling disorder is an important pathological feature of PE. Recent research indicates a strong correlation between the necroptosis of trophoblast cells and spiral artery remodeling in the development of PE [43].

Necroptosis is a novel type of programmed cell death that has been extensively investigated for its diagnostic and prognostic potential in various illnesses, such as ischemic cardiomyopathy [44], Stanford types A aortic dissection [45], and cutaneous melanoma [46]. In recent years, growing researchers have actively mined and analyzed data from GEO or other databases to find potential molecular markers for PE diagnosis and treatment. However, the role of necroptosis in PE remains to be elucidated, and studies of necroptosis combined with immune cell infiltration in PE are yet to be performed. Therefore, further research is warranted to fully elucidate the potential implications of necroptosis in PE pathophysiology.

Our study provides compelling evidence of the involvement of necroptosis and immune cell infiltration in the pathogenesis of PE. Specifically, we identified alterations in the expression of 9 NRDEGs, including BRAF, PAWR, USP22, SYNCRIP, KRT86, MERTK, BAP1, CXCL5, and STK38. Furthermore, GSEA revealed the activation of several necroptosis pathways. We then constructed a LASSO regression model using six NRDEGs, which demonstrated excellent diagnostic efficacy as reflected by ROC and DCA analyses. Additionally, we identified two distinct subtypes of PE, Cluster1 and Cluster2, based on the expression of key module genes. Among these genes, SYNCRIP, STK38, and KRT86 exhibited significant differences across the subtypes and displayed good diagnostic accuracy (AUC > 0.7). Remarkably, we also observed significant differences in the abundance of immune cells between the PE and control groups, as well as across both PE subtypes. Neutrophils and monocytes were strongly positively correlated with CXC15 but negatively correlated with SYNCRIP, while macrophages M2 displayed the opposite correlation with the two key module genes. PAWR exhibited the strongest negative correlation with macrophages, while CXCL5 was positively associated with macrophages in PE subtypes. To the best of our knowledge, this bioinformatics analysis is the first to demonstrate the involvement of necroptosis and immune infiltration in the pathogenesis of PE.

In this study, we intersected 175 DEGs (90 downregulated and 85 upregulated) acquired from combined databases with 623 NRGs identified from the GeneCards database and MSigDB. Then we screened 9 NRDEGs, including BRAF, PAWR, USP22, SYNCRIP, KRT86, MERTK, BAP1, CXCL5, and STK38. Among these NRDEGs, USP22 is involved in remodeling spiral arteries in the mouse placenta through multiple regulations, which affects the development of embryos [47], suggesting that the abnormal expression of USP22 might be involved in PE. The result supports the findings of our bioinformatic analysis. Subsequently, the enrichment study showed that NRDEGs were mainly engaged in immune-inflammatory response pathways, including chemokine signaling pathway, peptide hormone biosynthesis, apoptotic cleavage of cell adhesion, apoptosis, TNFR2 non-canonical NF-κB pathway, IL-17 signaling pathway, and trip mediated programmed cell death, etc.

A previous study showed that chemokines play a crucial role in the maternal–fetal interface during early pregnancy [48]. Expression of the chemokine receptor D6 is increased in PE trophoblast cells, but its functionality is reduced [49]. Additionally, the levels of IL-17 in serum and placental tissues of PE patients are increased [50]. High levels of IL-17 activate microvascular endothelial cells, cause a neutrophil inflammatory response, promote the increase of CXCL1 and CXCL2, and cause an excessive inflammatory response in PE patients [51]. These studies provide supporting the validity of the current study. In summary, our functional annotation and pathway enrichment analyses suggest that necroptosis and immunological inflammatory play a crucial role in the pathogenesis of PE.

The LASSO regression model was constructed, including 6 NRDEGs, namely BAP1, BRAF, KRT86, MERTK, PAWR, and USP22. This diagnostic model had good clinical utility by the calibration curve and DCA. BRAF and PAWR contributed more to the model than the other four genes by nomogram. In addition, six NRDEGs from the diagnostic model were used to calculate the N Score by ssGSEA. The ROC curve's AUC value indicated good effectiveness and predictability. By identifying NRDEGs in diagnostic model, our study provides a valuable starting point for further research in this area.

A total of 5 key gene modules, including MElightcyan, MEsalmon, MEpurple, MEbrown, and MEtan were screened out by correlation screening between WGCNA and N Score, and further intersecting the genes contained in these modules with 9 NRDEGs, six essential module genes (PAWR, SYNCRIP, MERTK, CXCL5, STK38, and KRT86) were obtained, which expression showed significant differences between PE and controls in combined datasets, the ROC curves of PAWR, SYNCRIP, CXCL5, and STK38 had higher AUC values in combined datasets. Finally, PE disease subtypes were identified using consensus clustering analysis based on the expression of six key module genes in PE samples. Two distinct disease subtypes were identified: subtype A (Cluster1 consisted of 17 samples) and subtype B (Cluster2 encompassed 43 samples). The expressions of SYNCRIP, STK38, and KRT86 in both subtypes of PE were significantly different, and their diagnostic accuracy among subtypes was relatively high (AUC > 0.7). Interestingly, the difference between the two PE subtypes is similar to that between PE and controls, apart from the MERTK gene. Especially for the genes in cluster 2, their expression patterns were similar to those in the control group. These interesting gene expression patterns suggest that different subtypes of PE may have differences in pathogenesis and pathophysiology. Similar to our clinical difference between early-onset and late-onset PE, subtype B tends to be hypertension induced by other diseases in the late trimester rather than placental dysfunction in the early trimester. In future studies, we need to explore further the expression patterns of these genes and their relationship between EOPE and LOPE.

These results suggest that five NRDEGs (PAWR, BRAF, SYNCRIP, CXCL5 and STK38) might be PE and PE subtypes diagnostic biomarkers. The CXCL family and related pathways, such as CXCL15, have also been demonstrated to play significant roles in PE [52], GDM [53], and RSA [54]. Furthermore, studies have shown that BRAF is essential for ERK activation and, embryonic development, placental vascular development [55]. Moreover, Wang et al. found that immunodeficiency caused by abnormal expression of BRAF, through the potential ceRNA network, regulates IL-10, TNF-α, IFN-γ and IL-10 levels downstream and participates in the pathophysiological of complicated pregnancy symptoms [56]. These are in accordance with the findings of this study. This research is the first to use PE-related NRDEGs to construct PE models of diagnostic and disease subtypes. However, we acknowledge that placental bed biopsy is not feasible during pregnancy. Our proposed diagnostic tool based on PE-related NRDEGs expression analysis must be further validated and refined before implementing it in clinical practice. This potential diagnostic study can serve as a starting point for developing more practical and non-invasive diagnostic analyses. In addition, this approach may provide insight into the underlying mechanisms of PE, which may facilitate the development of new treatment strategies.

In recent decades, growing evidence of immunological dysregulation in PE has emerged. For example, CD4 + memory T cell activation decreases [57], and the dysfunction of regulatory T cells affects the systemic immune responses [58]. However, the immune cell activation pattern in PE remains uncertain. We applied CIBERSORT to assess the immune infiltration comprehensively to identify the function of immune cell infiltration in PE. The score of most immune cells was lower in PE, and the findings were consistent with previous studies [59]. We discovered significant differences in the infiltration of monocytes, T cells follicular helper and regulatory T cells between PE and control groups. The infiltration of type 2 T helper cells, macrophages, and regulatory T cells was significantly different between cluster 1 and cluster 2. Macrophages and T cells are critical in regulating the immune system's equilibrium [60, 61]. These results further confirm that immune cell infiltration is significant for the pathogenesis and classification of PE. Necroptosis has been reported to regulate immune system components [62]. Thus, we examined the relationship between NRDEGs and the infiltration of immune cells in PE. We discovered that some NRDEGs had a moderate correlation with immune cells. Specifically, neutrophils and monocytes showed a moderate positive correlation with CXC15 and a moderate negative correlation with SYNCRIP. However, macrophages M2 showed an opposite correlation with the two key module genes in PE datasets. Additionally, further analysis shows that macrophage has the strongest negative correlation with PAWR and the strongest positive correlation with CXCL5 in both PE subtypes. These findings showed a moderate relationship between immune cell infiltrations and necroptosis, suggesting that necroptosis may promote the onset of PE by triggering immune infiltration and immunological response.

Our research has several areas for improvement. First, although we performed a thorough bioinformatics analysis in the present research, our findings still require cautious evaluation due to the lack of support from our experiments and clinical studies. Second, the large number of datasets might result in inevitable and removed batch differences during analysis. Third, different feature selection methods may produce different results, and this analysis focuses on the key module genes with the strongest correlation with the N score. However, the genes constituting the regression models are also valuable and will be considered in future studies to explore whether possible target genes influence the development and progression of PE.

##  Conclusions

Our bioinformatics investigation revealed substantial differences in the NRDEGs' expression levels between control and PE placenta samples. Moreover, we discovered a relationship between the NRDEGs' expression and several immune cells' infiltration in PE. The immunological and necroptosis-related factors found through our analysis may provide light on the pathophysiology of PE.

## Supplementary Information

**Additional file 1: Fig. S1.** GSVA for Combined Datasets. A-B. GSVA results in PE and control groups by group comparison boxplot (A) and complex heatmap (B). PE, Pre-eclampsia; GSVA, Gene Set Variation Analysis. Green represents the control group; pink represents the PE group. *: *p*-value < 0.05; **: *p*-value < 0.01; ***: *p*-value < 0.001. The screening criteria for GSEA were |logFC| > 0.25 and adj. *p*-value < 0.05.

**Additional file 2: Table S1.** Results of NRGs screening.

**Additional file 3: Table S2.** Results of GSVA for combined datasets.

## Data Availability

The datasets generated and analyzed during the current study are available in the Gene Expression Omnibus (GEO) database. (https://www.ncbi.nlm.nih.gov/geo/query/acc.cgi?acc=GSE60438).

## Abbreviations

PE: Preeclampsia
GO: Gene Ontology
KEGG: Kyoto Encyclopedia of Genes and Genomes
DEGs: Differentially expressed genes
NRDEGs: Necroptosis-related genes
PPI: Protein–protein interaction
PCA: Principal component analysis
CDF: Empirical Cumulative Distribution Function
ROC: Receiver Operating Characteristic
GSEA: Gene Set Enrichment Analysis
WGCNA: Weighted Correlation Network Analysis
LASSO: Least Absolute Shrinkage and Selection Operator
