# Supplementary figures and images for: Role of necroptosis and immune infiltration in preeclampsia: novel insights from bioinformatics analyses
Source: BMC Pregnancy Childbirth. 2023 Jul 4;23:495. doi: 10.1186/s12884-023-05821-0 (PMC10320970; doi:10.1186/s12884-023-05821-0)

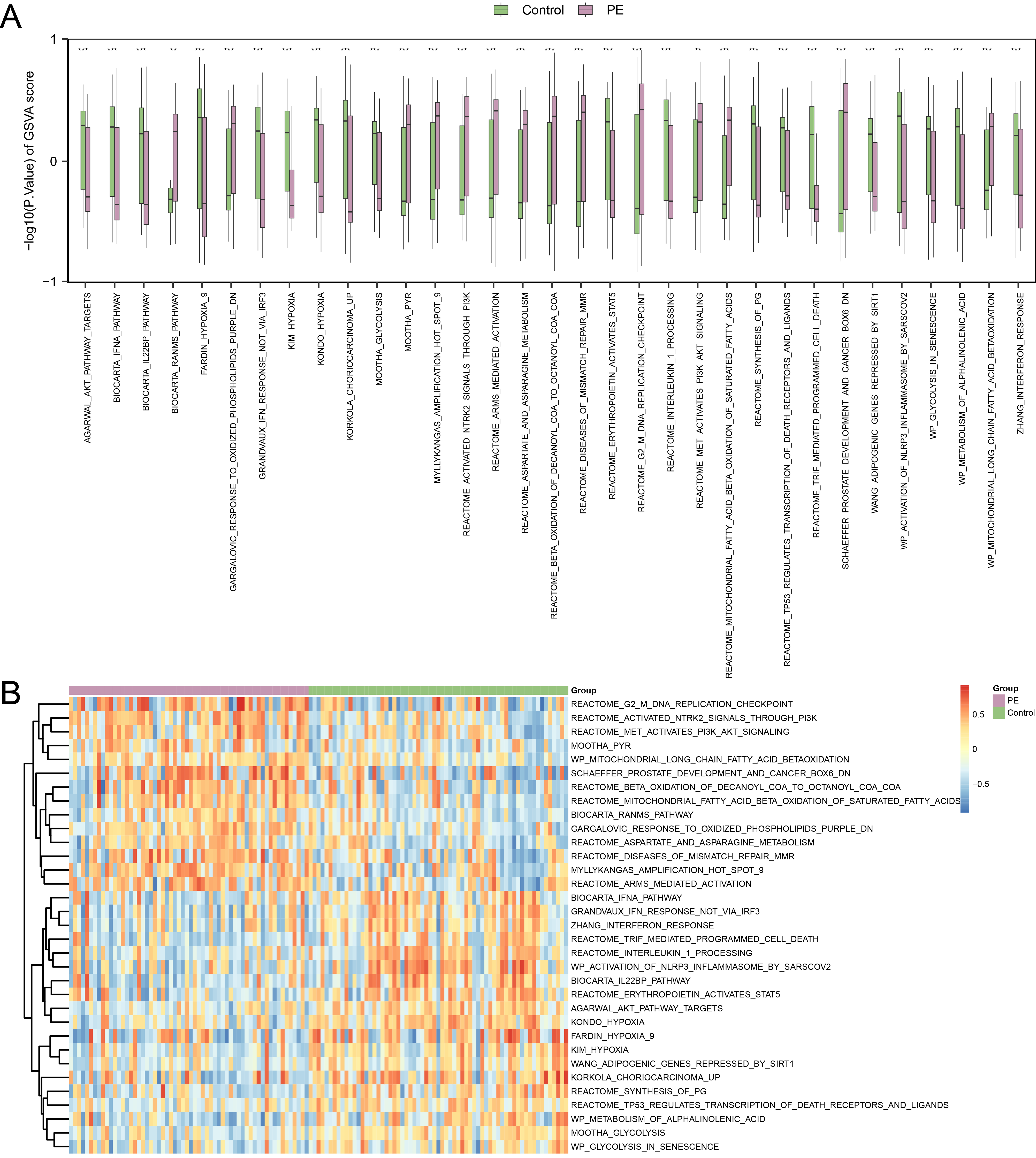

Supplement: Supplementary file 1 — Additional file 1: Fig. S1. GSVA for Combined Datasets. A-B. GSVA results in PE and control groups by group comparison boxplot (A) and complex heatmap (B). PE, Pre-eclampsia; GSVA, Gene Set Variation Analysis. Green represents the control group; pink represents the PE group. *: p-value < 0.05; **: p-value < 0.01; ***: p-value < 0.001. The screening criteria for GSEA were |logFC| > 0.25 and adj. p-value < 0.05. [file 12884_2023_5821_MOESM1_ESM.tif]
