# Supplementary material for: Role of necroptosis and immune infiltration in preeclampsia: novel insights from bioinformatics analyses
Source: BMC Pregnancy Childbirth. 2023 Jul 4;23:495. doi: 10.1186/s12884-023-05821-0 (PMC10320970; doi:10.1186/s12884-023-05821-0)
Supplement: Supplementary file 3 — Additional file 3: Table S2. Results of GSVA for combined datasets. [file 12884_2023_5821_MOESM3_ESM.doc]

**Table S2 Results of GSVA for Combined Datasets**

| **ID** | **logFC** | **AveExpr** | **p value** | **adj.p value** |
| --- | --- | --- | --- | --- |
| REACTOME_MITOCHONDRIAL_FATTY_ACID_BETA_OXIDATION_OF_SATURATED_FATTY_ACIDS | 3.68E-01 | -6.04E-03 | 9.93E-07 | 3.86E-03 |
| MOOTHA_GLYCOLYSIS | -2.87E-01 | -1.41E-02 | 2.71E-06 | 4.43E-03 |
| KONDO_HYPOXIA | -3.28E-01 | 5.08E-02 | 3.37E-06 | 4.43E-03 |
| REACTOME_ACTIVATED_NTRK2_SIGNALS_THROUGH_PI3K | 3.47E-01 | 2.60E-02 | 3.48E-06 | 4.43E-03 |
| AGARWAL_AKT_PATHWAY_TARGETS | -2.80E-01 | 1.36E-02 | 1.92E-05 | 1.59E-02 |
| MYLLYKANGAS_AMPLIFICATION_HOT_SPOT_9 | 3.38E-01 | 4.76E-02 | 2.25E-05 | 1.59E-02 |
| REACTOME_SYNTHESIS_OF_PG | -3.16E-01 | -1.80E-02 | 2.25E-05 | 1.59E-02 |
| WP_MITOCHONDRIAL_LONG_CHAIN_FATTY_ACID_BETAOXIDATION | 2.70E-01 | 2.20E-02 | 2.93E-05 | 1.64E-02 |
| KIM_HYPOXIA | -2.98E-01 | -3.77E-02 | 3.61E-05 | 1.64E-02 |
| REACTOME_TP53_REGULATES_TRANSCRIPTION_OF_DEATH_RECEPTORS_AND_LIGANDS | -2.59E-01 | 1.43E-03 | 4.14E-05 | 1.68E-02 |
| REACTOME_ARMS_MEDIATED_ACTIVATION | 3.30E-01 | 2.71E-02 | 4.50E-05 | 1.68E-02 |
| GARGALOVIC_RESPONSE_TO_OXIDIZED_PHOSPHOLIPIDS_PURPLE_DN | 2.84E-01 | 1.76E-02 | 4.82E-05 | 1.70E-02 |
| REACTOME_BETA_OXIDATION_OF_DECANOYL_COA_TO_OCTANOYL_COA_COA | 3.48E-01 | 2.85E-03 | 5.39E-05 | 1.81E-02 |
| REACTOME_ERYTHROPOIETIN_ACTIVATES_STAT5 | -3.14E-01 | -6.98E-03 | 6.75E-05 | 1.81E-02 |
| KORKOLA_CHORIOCARCINOMA_UP | -3.47E-01 | 1.43E-02 | 6.78E-05 | 1.81E-02 |
| REACTOME_INTERLEUKIN_1_PROCESSING | -2.97E-01 | -1.06E-02 | 6.83E-05 | 1.81E-02 |
| WANG_ADIPOGENIC_GENES_REPRESSED_BY_SIRT1 | -2.55E-01 | -3.60E-02 | 8.46E-05 | 1.82E-02 |
| BIOCARTA_RANMS_PATHWAY | 2.62E-01 | -8.69E-02 | 1.04E-04 | 2.14E-02 |
| BIOCARTA_IL22BP_PATHWAY | -3.00E-01 | -5.17E-02 | 1.67E-04 | 2.95E-02 |
| WP_ACTIVATION_OF_NLRP3_INFLAMMASOME_BY_SARSCOV2 | -3.22E-01 | 1.75E-02 | 1.97E-04 | 3.33E-02 |
| SCHAEFFER_PROSTATE_DEVELOPMENT_AND_CANCER_BOX6_DN | 3.58E-01 | 2.58E-03 | 2.09E-04 | 3.41E-02 |
| WP_METABOLISM_OF_ALPHALINOLENIC_ACID | -2.87E-01 | -1.01E-01 | 2.42E-04 | 3.84E-02 |
| ZHANG_INTERFERON_RESPONSE | -2.73E-01 | -1.92E-02 | 2.65E-04 | 4.00E-02 |
| REACTOME_TRIF_MEDIATED_PROGRAMMED_CELL_DEATH | -2.71E-01 | -1.11E-01 | 2.92E-04 | 4.00E-02 |
| REACTOME_G2_M_DNA_REPLICATION_CHECKPOINT | 3.55E-01 | 2.13E-02 | 2.96E-04 | 4.00E-02 |
| MOOTHA_PYR | 2.70E-01 | -5.28E-02 | 3.03E-04 | 4.02E-02 |
| FARDIN_HYPOXIA_9 | -3.42E-01 | -1.05E-02 | 3.17E-04 | 4.02E-02 |
| REACTOME_ASPARTATE_AND_ASPARAGINE_METABOLISM | 2.61E-01 | -5.78E-02 | 3.88E-04 | 4.04E-02 |
| GRANDVAUX_IFN_RESPONSE_NOT_VIA_IRF3 | -2.72E-01 | -4.20E-02 | 4.50E-04 | 4.04E-02 |
| REACTOME_DISEASES_OF_MISMATCH_REPAIR_MMR | 3.04E-01 | 1.52E-02 | 4.53E-04 | 4.04E-02 |
| WP_GLYCOLYSIS_IN_SENESCENCE | -2.54E-01 | -6.01E-02 | 4.70E-04 | 4.04E-02 |
| REACTOME_MET_ACTIVATES_PI3K_AKT_SIGNALING | 2.68E-01 | 1.76E-02 | 6.46E-04 | 4.49E-02 |
| BIOCARTA_IFNA_PATHWAY | -2.65E-01 | -1.92E-02 | 6.47E-04 | 4.49E-02 |
| REACTOME_ABACAVIR_METABOLISM | -5.01E-01 | 2.41E-02 | 7.59E-04 | 3.49E-02 |
| REACTOME_DEFECTIVE_GALNT3_CAUSES_HFTC | -5.14E-01 | 7.93E-02 | 7.86E-04 | 3.52E-02 |
| OHASHI_AURKA_TARGETS | 5.28E-01 | 3.58E-02 | 7.89E-04 | 3.52E-02 |
| SCHAEFFER_PROSTATE_DEVELOPMENT_AND_CANCER_BOX2_DN | -5.23E-01 | -5.32E-02 | 9.15E-04 | 3.84E-02 |
| CHESLER_BRAIN_D6MIT150_QTL_CIS | 5.33E-01 | 1.56E-02 | 1.18E-03 | 4.29E-02 |
| WALLACE_PROSTATE_CANCER_DN | 5.01E-01 | -1.00E-02 | 1.35E-03 | 4.59E-02 |
| WP_ULTRACONSERVED_REGION_339_MODULATION_OF_TUMOR_SUPPRESSOR_MICRORNAS_IN_CANCER | 6.44E-01 | -2.73E-02 | 1.46E-03 | 4.86E-02 |

GSVA, Gene Set Variation Analysis.
